# ARMC5-CUL3 E3 ligase targets full-length SREBF in adrenocortical tumors

**DOI:** 10.1172/jci.insight.151390

**Published:** 2022-08-22

**Authors:** Yosuke Okuno, Atsunori Fukuhara, Michio Otsuki, Iichiro Shimomura

**Affiliations:** 1 Department of Metabolic Medicine and; 2 Department of Adipose Management, Osaka University Graduate School of Medicine, Osaka, Japan.

**Keywords:** Endocrinology, Cholesterol, Oncogenes, Ubiquitin-proteosome system

## Abstract

Inactivating mutations of *ARMC5* are responsible for the development of bilateral macronodular adrenal hyperplasia (BMAH). Although ARMC5 inhibits adrenocortical tumor growth and is considered a tumor-suppressor gene, its molecular function is poorly understood. In this study, through biochemical purification using SREBF (SREBP) as bait, we identified the interaction between SREBF and ARMC5 through its Armadillo repeat. We also found that ARMC5 interacted with CUL3 through its BTB domain and underwent self-ubiquitination. ARMC5 colocalized with SREBF1 in the cytosol and induced proteasome-dependent degradation of full-length SREBF through ubiquitination. Introduction of missense mutations in Armadillo repeat of ARMC5 attenuated the interaction between SREBF, and introduction of mutations found in BMAH completely abolished its ability to degrade full-length SREBF. In H295R adrenocortical cells, silencing of *ARMC5* increased full-length SREBFs and upregulated SREBF2 target genes. siARMC5-mediated cell growth was abrogated by simultaneous knockdown of *SREBF2* in H295R cells. Our results demonstrate that ARMC5 was a substrate adaptor protein between full-length SREBF and CUL3-based E3 ligase, and they suggest the involvement of the SREBF pathway in the development of BMAH.

## Graphical abstract

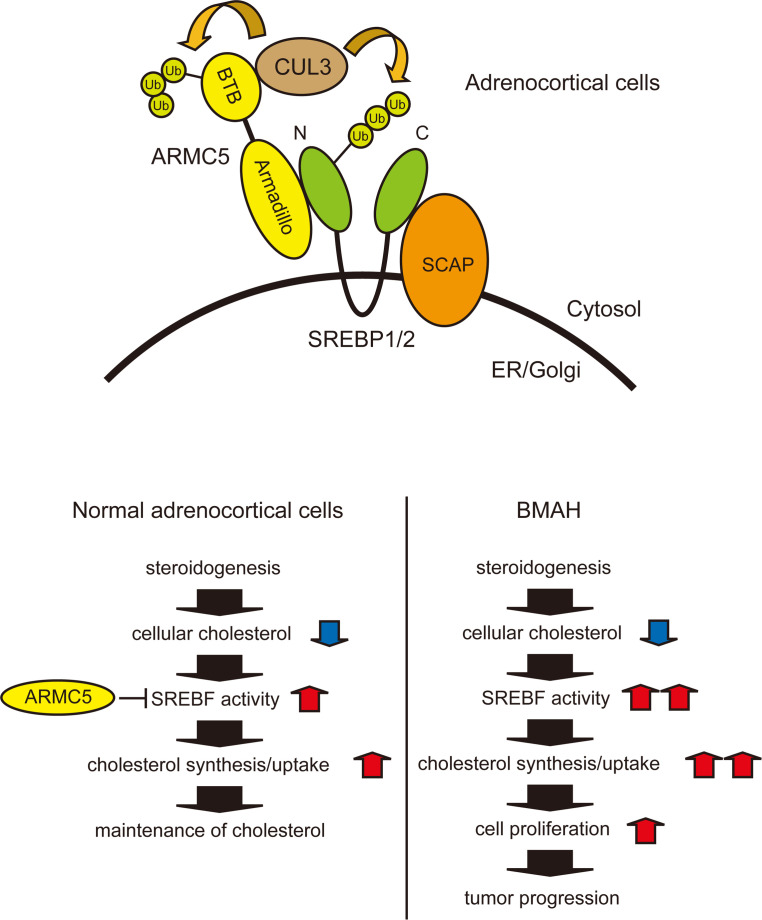

## Introduction

The causes of adrenal Cushing syndrome are largely divided into 3 categories: unilateral cortisol-producing adenoma, primary pigmented nodular adrenocortical disease (PPNAD), and bilateral macronodular adrenal hyperplasia (BMAH). The majority of the causal genes of the former 2 categories are involved in activation of cyclic AMP/protein kinase A (cAMP/PKA) pathway (1), such as *PRKACA* (2–5), *PRKAR1A* (6), *GNAS1* (7), *PDE11A* (8), and *PDE8B* (2). The molecular mechanism of these genes in adrenal Cushing syndrome is comprehensible because cAMP/PKA pathway is the downstream of adrenocorticotropic hormone (ACTH), stimulating cell growth and cortisol synthesis in adrenocortical cells.

In case of BMAH, inactivating mutations of *ARMC5* are responsible for approximately half of cases (9). *ARMC5* is considered a tumor-suppressor gene, as overexpression of *ARMC5* induced apoptosis (9, 10), whereas knockdown of *ARMC5* increased proliferating capacity with upregulation of cyclin E (*CCNE1*) (10) in adrenocortical cells. However, contrary to the genes involved in cAMP/PKA pathway, its molecular mechanism was largely unknown. ARMC5 contains 2 protein interaction domains, Armadillo repeat and a broad-complex, tramtrack, and bric a brac (BTB) domain (11). Recently, Cavalcante et al. reported the interaction between ARMC5 and CUL3, a component of ubiquitin E3 ligase complex, through BTB domain. While they showed that ARMC5 was self-ubiquitinated through a CUL3-dependent mechanism, they also noted the possibility that ARMC5 might be an adaptor protein between yet-unknown substrate and CUL3-based ubiquitin ligase (12).

SREBFs (also known as SREBPs) are encoded by 2 different genes, *SREBF1* and *SREBF2*. SREBFs are synthesized as unactivated full-length SREBFs attached to the endoplasmic reticulum (ER). Under depletion of cellular cholesterol, SREBFs are transported to the Golgi, where N-terminal fragment is cleaved by 2 proteases and is transported to the nucleus. Then, nuclear SREBFs work as transcription factors and upregulate genes related to cholesterol synthesis and lipogenesis. In addition to the well-known roles in lipid metabolism, such as fatty liver and circulating cholesterol, SREBFs are frequently activated in cancer cells, which must produce enough cholesterol to synthesize new membranes for replication (13).

In this study, we investigate the interactor of SREBF in adipocytes, because we recently reported that the elimination of oxidative stress in adipocytes improved insulin resistance with increased adiposity through modulation of SREBF1 activity (14). However, we found that ARMC5 was an adaptor between full-length SREBF and CUL3-based E3 ligase, and we elucidated its functional relationship in the development of BMAH.

## Results

**Figure 1:**
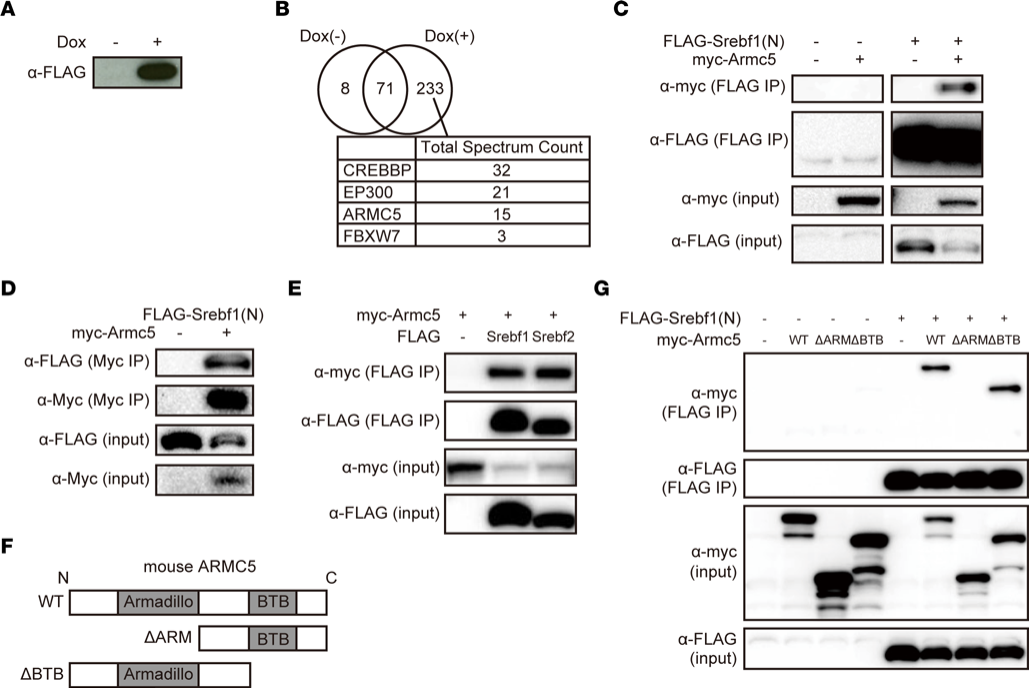
ARMC5 interacted with N-terminus of SREBFs through the Armadillo repeat. (**A**) Western blotting of lysates from the differentiated 3T3-L1–*TetON-FLAG-Srebf1(N)* cells treated with or without 10 μg/mL doxycycline for 30 hours with an anti-FLAG antibody. (**B**) Venn diagrams representing the number of identified proteins by LC-MS/MS of the samples immunoprecipitated with anti-FLAG antibody from the nuclear extracts of the differentiated 3T3-L1–*TetON-FLAG-Srebf1(N)* cells treated with or without 10 μg/mL doxycycline. Total spectrum counts of LC-MS/MS of the indicated protein in the sample treated with doxycycline are shown. (**C**) Western blotting of lysates (input) or samples immunoprecipitated with anti-FLAG antibody (FLAG IP) from the HEK293T cells transfected with pcDNA3.1-*FLAG*, pcDNA3.1-*myc*, pcDNA3.1-*FLAG-Srebf1(N)*, and/or pcDNA3.1-*myc-mArmc5* with the indicated antibodies. (**D**) Western blotting of lysates (input) or samples immunoprecipitated with anti-myc antibody (Myc IP) from the HEK293T cells transfected with pcDNA3.1-*myc*, pcDNA3.1-*myc-mArmc5*, and pcDNA3.1-*FLAG-Srebf1(N)* with the indicated antibodies. (**E**) Western blotting of lysates (input) or samples immunoprecipitated with anti-FLAG antibody (FLAG IP) from the HEK293T cells transfected with pcDNA3.1-*FLAG*, pcDNA3.1-*FLAG-Srebf1(N)*, pcDNA3.1-*FLAG-Srebf2(N)*, and/or pcDNA3.1-*myc-mArmc5* with the indicated antibodies. (**F**) Schematic representation of the structure of mouse ARMC5 (WT), the N-terminal deletion mutant (ΔARM), and the C-terminal deletion mutant (ΔBTB). (**G**) Western blotting of lysates (input) or samples immunoprecipitated with anti-FLAG antibody (FLAG IP) from the HEK293T cells transfected with pcDNA3.1-*FLAG*, pcDNA3.1-*myc,* pcDNA3.1-*FLAG-Srebf1(N)*, pcDNA3.1-*myc-mArmc5* (WT), pcDNA3.1-*myc-mArmc5ΔARM* (ΔARM), and/or pcDNA3.1-*myc-mArmc5ΔBTB* (ΔBTB) with the indicated antibodies. See complete unedited blots in the supplemental material.

To explore the regulator of SREBF1 in adipocytes, we performed biochemical purification using N-terminus of SREBF1 (SREBF1[N]) as bait. Differentiated 3T3-L1 adipocytes stably introduced with *TetON-FLAG-Srebf1(N)* expressed FLAG-SREBF1(N) in a doxycycline-dependent manner (Figure 1A). Extracts of these cells were immunoprecipitated with an anti-FLAG antibody and analyzed by liquid chromatography–tandem mass spectrometry (LC-MS/MS). Among 233 proteins specific to doxycycline-treated cells, ARMC5 was identified together with the known interactors of SREBF(N), such as CREBBP (15), EP300 (15), and FBXW7 (16) (Figure 1B). Coimmunoprecipitation confirmed the interaction between SREBF1(N) and mouse ARMC5 (Figure 1, C and D). ARMC5 similarly interacted with N-terminus of SREBF2 (SREBF2[N]) (Figure 1E). ARMC5 harbors an N-terminal Armadillo repeat and a C-terminal BTB domain (Figure 1F). SREBF1(N) interacted with the C-terminal deletion mutant (ARMC5ΔBTB) but not the N-terminal deletion mutant (ARMC5ΔARM) of ARMC5 (Figure 1G). These data indicate that ARMC5 interacted with N-terminus of SREBFs through its Armadillo repeat.

Although we had started the experiments with interest of SREBF1 in adipocytes, we switched our focus to the adrenocortical cells. This was because (a) *ARMC5* was the causal gene of BMAH, the hyperplasia of adrenocortical cells (9); (b) gene expression of *SREBF1* and *SREBF2* are the highest or the second highest in adrenal gland (Supplemental Figure 2A; supplemental material available online with this article; https://doi.org/10.1172/jci.insight.151390DS1) or adrenal cortex (Supplemental Figure 2B) among tissues including adipocytes; and (c) steroids were synthesized from cholesterol, and SREBF is the master regulator of cholesterol metabolism.

**Figure 2:**
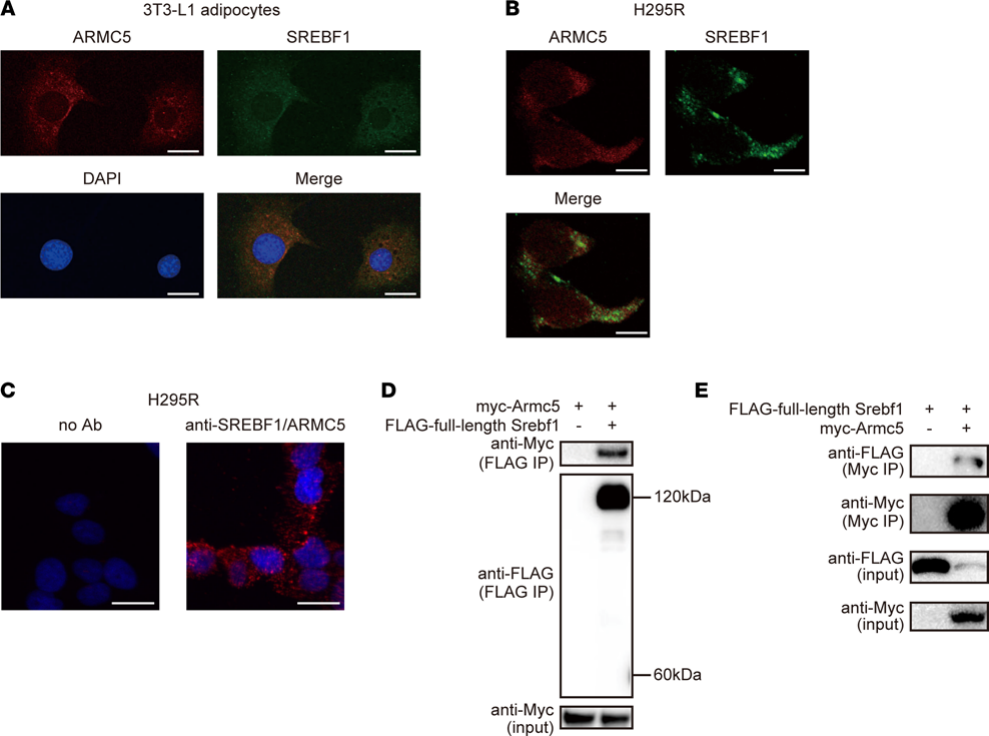
Colocalization of ARMC5 and SREBF1 in the cytosol. (**A** and **B**) Confocal microscopy of differentiated 3T3-L1 adipocytes (**A**) or NCI-H295R adrenocortical cells (**B**) costained with Texas Red anti-ARMC5 (red), DyLight 488 anti-SREBF1 (green), and DAPI (blue). Scale bar: 20 μm. (**C**) In situ proximity ligation assay (red) in NCI-H295R cells without antibody (no Ab) or with anti-SREBF1 antibody and anti-ARMC5 antibody (α-SREBF1/ARMC5) costained with DAPI (blue). Scale bar: 20 μm. (**D**) Western blotting of lysates (input) or samples immunoprecipitated with an anti-FLAG antibody (FLAG IP) from the HEK293T cells transfected with pcDNA3.1-*FLAG* or pcDNA3.1-*FLAG-Srebf1* together with pcDNA3.1-*myc-mArmc5* with the indicated antibodies. (**E**) Western blotting of lysates (input) or samples immunoprecipitated with anti-myc antibody (Myc IP) from the HEK293T cells transfected with pcDNA3.1-*myc* or pcDNA3.1-*myc-mArmc5* together with pcDNA3.1-*FLAG-Srebf1* with indicated antibodies. See complete unedited blots in the supplemental material.

The N-terminus of SREBF can exist in 2 forms in distinct subcellular compartments. One is the cytosol, where N-terminus of full-length SREBF is projected. The other is the nucleus, where cleaved N-terminus of SREBF works as transcription factor. To elucidate the subcellular compartment of the interaction site of ARMC5 and SREBF, we employed an immunocytochemical approach. We found that ARMC5 was mainly localized in the cytosol, consistent with recent reports (17, 18), and the majority of ARMC5 and SREBF1 were colocalized in the cytosol in 3T3-L1 adipocytes (Figure 2A) and H295R adrenocortical cells (Figure 2B). The specificity of immunodetection was verified by knockdown of each protein (Supplemental Figure 1). Close proximity between ARMC5 and SREBF1 was verified by in situ proximity ligation assay in H295R cells (Figure 2C). In fact, ARMC5 was coimmunoprecipitated with full-length SREBF1 (Figure 2D) (note that the estimated 60 kDa band of FLAG-nuclear SREBF1 cleaved from FLAG full-length SREBF1 was negligible). The interaction between ARMC5 and full-length SREBF1 was also verified by reciprocal IP (Figure 2E) (note that coexpression of myc-ARMC5 drastically decreased the protein amount of FLAG full-length SREBF1). Based on these data, ARMC5 was shown to potentially interact with N-terminus of full-length SREBF1 in the cytosol.

**Figure 3:**
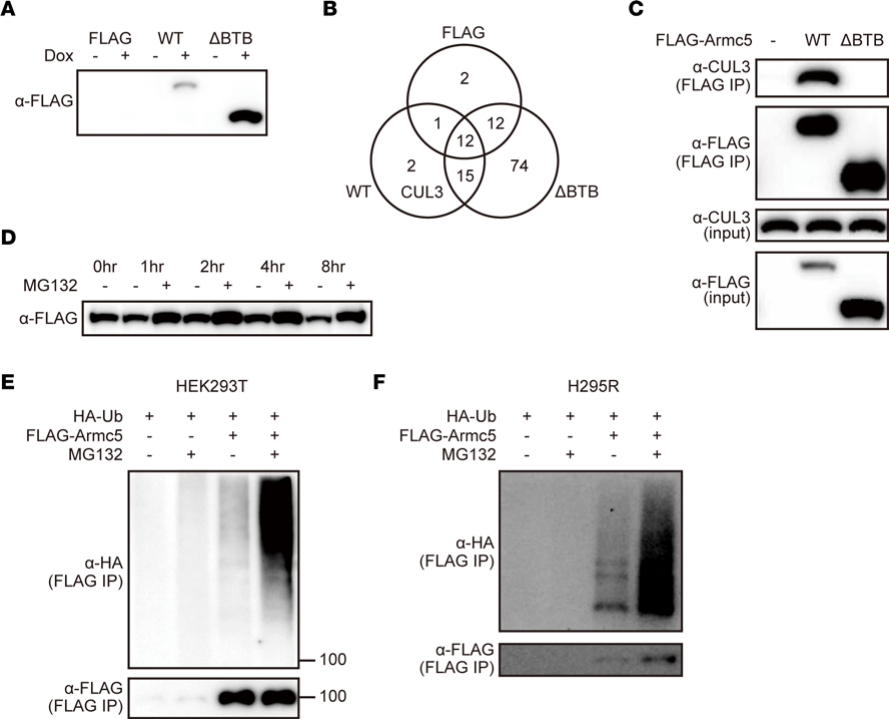
ARMC5 interacted with CUL3 and underwent self-ubiquitination. (**A**) Western blotting of lysates from the differentiated 3T3-L1*–TetON-FLAG* (FLAG), 3T3-L1–*TetON-FLAG-Armc5* (WT), or 3T3-L1–*TetON-FLAG-Armc5ΔBTB* (ΔBTB) cells treated with or without 10 μg/mL doxycycline for 30 hours with an anti-FLAG antibody. (**B**) Venn diagrams representing the number of identified proteins by LC-MS/MS of the sample immunoprecipitated with anti-FLAG antibody from the nuclear extracts of the differentiated 3T3-L1–*TetON-FLAG* (FLAG), 3T3-L1–*TetON-FLAG-Armc5* (WT), or 3T3-L1–*TetON-FLAG-Armc5ΔBTB* (ΔBTB) cells treated with 10 μg/mL doxycycline. (**C**) Western blotting of lysates (input) or samples immunoprecipitated with high-salt TNE buffer and anti-FLAG antibody (IP) from the HEK293T cells transfected with pcDNA3.1-*FLAG*, pcDNA3.1-*FLAG-mArmc5* (WT), or pcDNA3.1-*FLAG-mArmc5ΔBTB* (ΔBTB) with the indicated antibodies. (**D**) Western blotting of lysates from the HEK293T cells transfected with pcDNA3.1-*FLAG-mArmc5* for 24 hours, followed by treatment with 10 μM MG132 for the indicated times with an anti-FLAG antibody. (**E** and **F**) Cell-based ubiquitination assays of the HEK293T cells (**E**) or H295R cells (**F**) transfected with pcDNA3.1-*FLAG* or pcDNA3.1-*FLAG-mArmc5*, followed by treatment with or without MG132, using the indicated antibody. See complete unedited blots in the supplemental material.

We next sought the interactors of the BTB domain of ARMC5 by biochemical purification. 3T3-L1 cells stably introduced with *TetON-FLAG*, *TetON-FLAG-Armc5*, or *TetON-FLAG-Armc5ΔBTB* were differentiated into adipocytes that expressed these proteins in a doxycycline-dependent manner (Figure 3A). Extracts of these cells treated with doxycycline were immunoprecipitated with an anti-FLAG antibody and analyzed by LC-MS/MS. CUL3, which is known to associate with multiple BTB domain–containing proteins (19), was identified in the 3T3-L1–*TetON-FLAG-Armc5* cells but not in 3T3-L1–*TetON-FLAG* or 3T3-L1–*TetON-FLAG-Armc5ΔBTB* (Figure 3B). Coimmunoprecipitation confirmed the interaction between CUL3 and ARMC5 but not ARMC5ΔBTB (Figure 3C). Inhibition of the proteasome pathway using MG132 resulted in the accumulation of FLAG-ARMC5 protein in HEK293T cells (Figure 3D), as well as ubiquitinated FLAG-ARMC5 in HEK293T cells (Figure 3E) and H295R cells (Figure 3F). Our comprehensive approach recapitulated the recent findings that ARMC5 interacted with CUL3 and was self-ubiquitinated and degraded through a CUL3-dependent mechanism (12). Concomitantly, these data raised the possibility that ARMC5 was an adaptor protein between full-length SREBF and CUL3-based E3 ligase.

**Figure 4:**
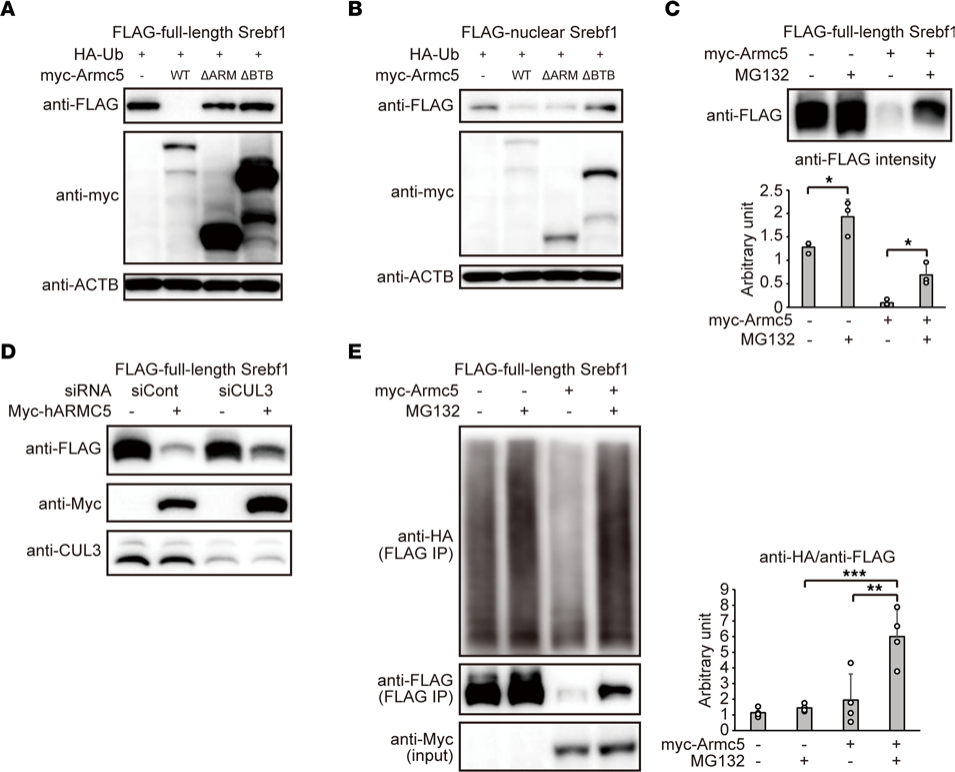
ARMC5 ubiquitinated and degraded full-length SREBF1. (**A**) Western blotting of lysates from the HEK293T cells transfected with pcDNA3.1-*FLAG-Srebf1* and pRK5-HA-Ubiquitin-WT, together with pcDNA3.1-*myc* (–), pcDNA3.1-*myc-mArmc5* (WT), pcDNA3.1-*myc-mArmc5ΔARM* (ΔARM), or pcDNA3.1-*myc-mArmc5ΔBTB* (ΔBTB) for 24 hours with the indicated antibody. (**B**) Western blotting of lysates from the HEK293T cells transfected with pcDNA3.1-*FLAG-Srebf1(N)* and pRK5-HA-Ubiquitin-WT, together with pcDNA3.1-*myc* (–), pcDNA3.1-*myc-mArmc5* (WT), pcDNA3.1-*myc-mArmc5ΔARM* (ΔARM), or pcDNA3.1-*myc-mArmc5ΔBTB* (ΔBTB) for 24 hours with the indicated antibody. (**C**) Western blotting of lysates from the HEK293T cells transfected with pcDNA3.1-*FLAG-Srebf1* together with pcDNA3.1-*myc* (–) or pcDNA3.1-*myc-mArmc5* treated with or without 10 μM MG132 with the indicated antibodies. The density of multiple experiments were quantified (bottom) (*n* = 3, each). (**D**) Western blotting of lysates from HEK293T cells transfected with negative control siRNA (siCont) or siRNA targeting to CUL3 (siCUL3) followed by transfection of pcDNA3.1-*myc* or pcDNA3.1-*myc-hARMC5* together with pcDNA3.1-*FLAG-Srebf1* with indicated antibodies. (**E**) Cell-based ubiquitination assay of the HEK293T cells transfected with pcDNA3.1-*FLAG-Srebf1* together with pcDNA3.1-*myc* (–) or pcDNA3.1-*myc-mArmc5* (+), followed by treatment with or without MG132, using the indicated antibody. The density of anti-HA relative to anti-FLAG were quantified (right) (*n* = 4, each). *P < 0.05, **P < 0.01, ***P < 0.001 by Tukey-Kramer test. See complete unedited blots in the supplemental material.

In accordance with this hypothesis, overexpression of *Armc5* drastically reduced the protein amount of full-length SREBF1, and such reduction was not observed in overexpression of *Armc5ΔARM* or *Armc5ΔBTB* (Figure 4A). In contrast, overexpression of *Armc5* decreased, to a minor extent, protein amount of nuclear SREBF1, and such a change was similarly seen in overexpression of *Armc5ΔARM* (Figure 4B). The reduction of full-length SREBF1 by overexpression of *Armc5* was rescued by treatment with proteasome inhibitor MG132 (Figure 4C) or by knockdown of *CUL3* (Figure 4D). Ubiquitination of full-length SREBF1 was augmented by overexpression of *Armc5* (Figure 4E). Collectively, we concluded that ARMC5 was a substrate adaptor protein between full-length SREBF and CUL3-based E3 ligase that facilitated ubiquitination and degradation.

**Figure 5:**
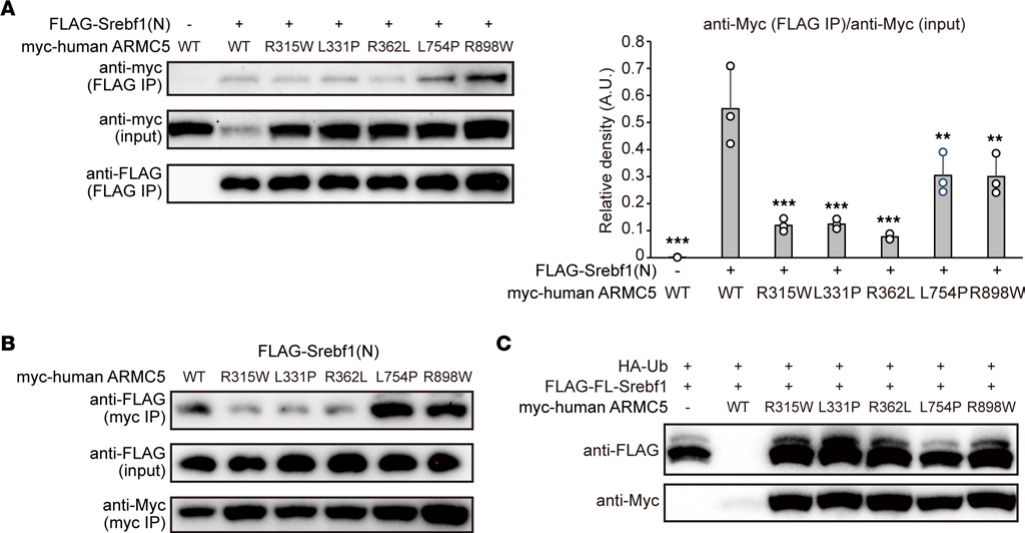
ARMC5 mutation in BMAH abrogated the interaction with SREBF1. (**A**) Western blotting of lysates (input) or samples immunoprecipitated with an anti-FLAG antibody (FLAG IP) from the HEK293T cells transfected with pcDNA3.1-*FLAG*, pcDNA3.1-*FLAG-Srebf1(N)*, pcDNA3.1-*myc-hARMC5* (WT), and/or pcDNA3.1-*myc-hARMC5* harboring indicated mutation with the indicated antibodies. The density of anti-myc (FLAG IP) relative to anti-myc (input) of multiple experiments were quantified (right) (*n* = 3, each). **P < 0.01, **P < 0.001 compared with WT by Dunnett’s test. (**B**) Western blotting of lysates (input) or samples immunoprecipitated with anti-myc antibody (myc IP) from HEK293T cells transfected with pcDNA3.1-*myc-hARMC5* (WT) or pcDNA3.1-*myc-hARMC5* harboring indicated mutation together with pcDNA3.1-*FLAG-Srebf1(N)* with indicated antibodies. (**C**) Western blotting of lysates from the HEK293T cells transfected with pcDNA3.1-*FLAG-Srebf1* and pRK5-HA-Ubiquitin-WT, together with pcDNA3.1-*myc* (–), pcDNA3.1-*myc-mArmc5* (WT), or pcDNA3.1-*myc-hArmc5* harboring indicated mutation for 24 hours with the indicated antibody. See complete unedited blots in the supplemental material.

Mutations of *ARMC5* are responsible for BMAH (9). The majority of *ARMC5* mutations result in loss of heterozygosity, nonsense mutation, or frameshift, which all lead to complete loss of ARMC5 protein. Several missense mutations had also been identified in BMAH, and these were supposed to be loss-of-function mutations. Similarly seen in mouse ARMC5 (Figure 1), SREBF1 interacted with human ARMC5, and introduction of R315W, L331P, and R362L mutation to human ARMC5 (located in the Armadillo repeat), drastically attenuated the interaction with SREBF1, which was less prominent in L754P mutant (located in the BTB domain) or R898W mutant (located outside these domains) (Figure 5, A and B). All of the 5 mutants (R315W, L331P, R362L, L754P, and R898W) completely lost their ability to reduce protein amount of full-length SREBF1 (Figure 5C). These data implicate the importance of ARMC5-mediated degradation of full-length SREBFs in the development of BMAH.

**Figure 6:**
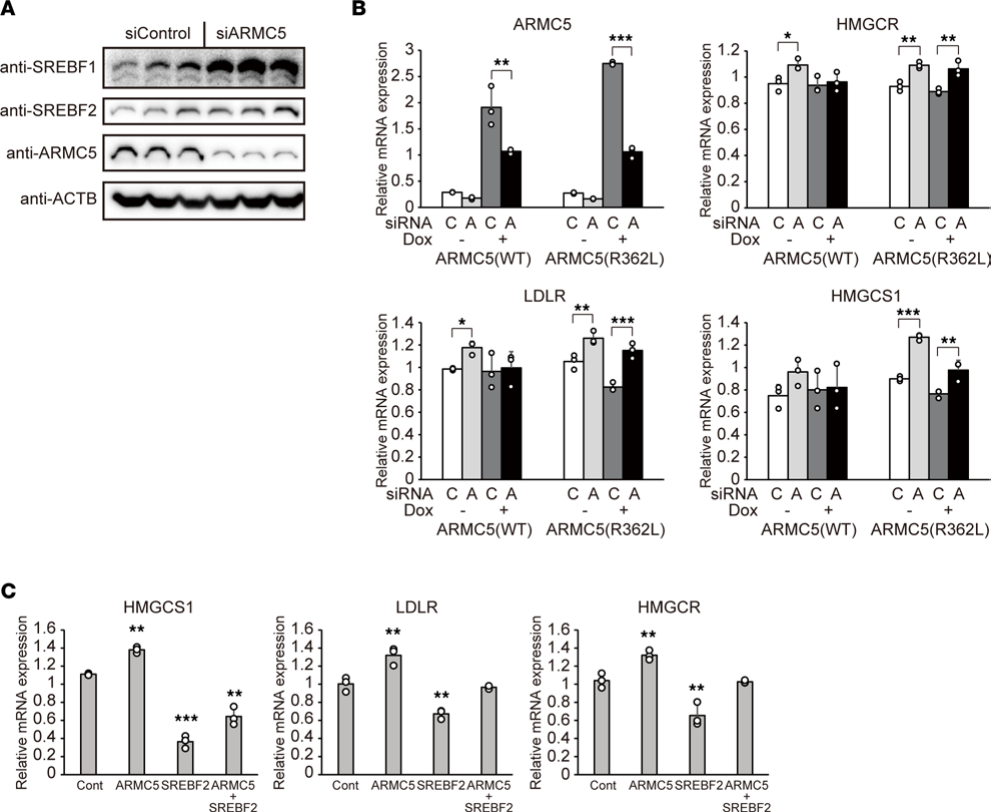
Regulation of SREBF2 by ARMC5 in H295R cells. (**A**) Western blotting of lysates from H295R cells transfected with negative control siRNA (siControl) or siRNA targeting to ARMC5 (siARMC5) for 48 hours with indicated antibodies. (**B**) Gene expression of the indicated genes in H295R-*TetON-hARMC5* (ARMC5[WT]) or H295R-*TetON-hARMC5(R362L)* (ARMC5[R362L]) transfected with negative control siRNA (C) or siRNA targeting to ARMC5 (A) for 48 hours followed by treatment of doxycycline for 48 hours (*n* = 3, each). *P < 0.05, **P < 0.01, ***P < 0.001 by Tukey-Kramer test. (**C**) Gene expression of the indicated genes in H295R cells transfected with negative control siRNA (Cont), siRNA targeting to ARMC5 (ARMC5), and/or siRNA targeting to SREBF2 (SREBF2) for 72 hours (*n* = 3, each). **P < 0.01, ***P < 0.001 by Dunnett’s test, compared with siControl. See complete unedited blots in the supplemental material.

To elucidate the roles of ARMC5 in adrenocortical cells, siRNA targeting to *ARMC5* was introduced in H295R human adrenocortical cells. Silencing of *ARMC5* increased protein amount of full-length SREBF1 and SREBF2 (Figure 6A). Knockdown of *ARMC5* significantly upregulated cholesterol-related genes, such as *HMGCS1*, *HMGCR*, and *LDLR*, and these changes were abolished by doxycycline-mediated expression of *ARMC5*, but not by that of *ARMC5(R362L)* (Figure 6B). In general, SREBF1 is mainly involved in lipogenesis, and SREBF2 is mainly involved in cholesterol metabolism (20). In H295R adrenocortical cells, cholesterol-related genes were also regulated by SREBF2, as siSREBF2 significantly downregulated cholesterol-related genes, such as *HMGCS1* and *HMGCR*, while siSREBF1 had no effect on the expression of these genes (Supplemental Figure 3). The siARMC5-mediated upregulation of cholesterol-related genes was attenuated by simultaneous silencing of *SREBF2* (Figure 6C and Supplemental Figure 4). These findings indicate that endogenous ARMC5 decreased protein amount of full-length SREBF and suppressed SREBF2-mediated gene expression of cholesterol-related genes.

**Figure 7:**
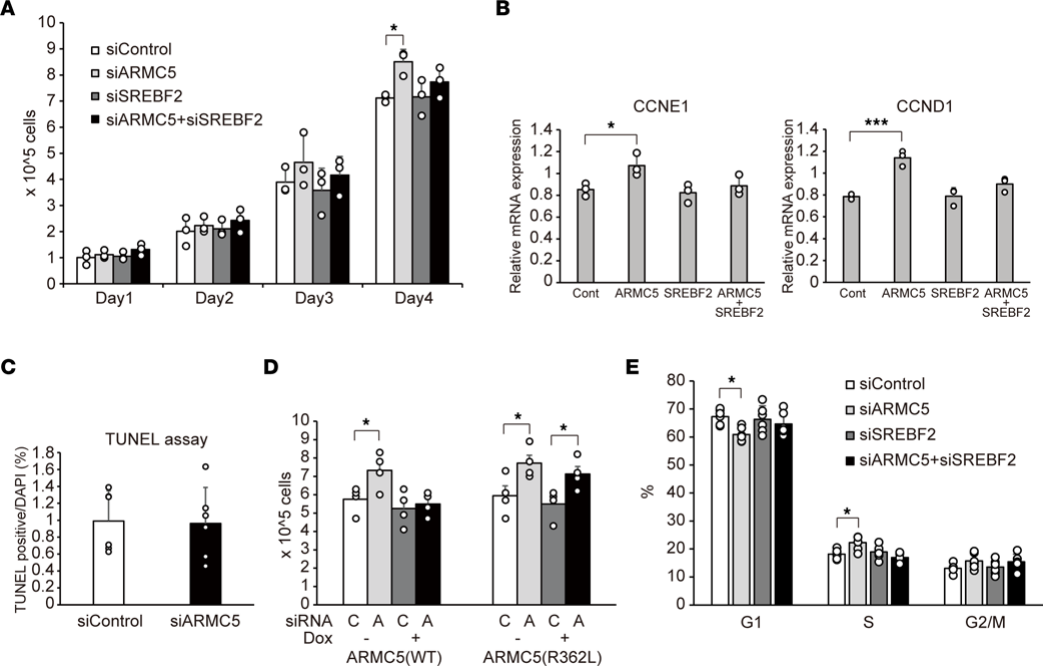
siARMC5-mediated cell growth was dependent on SREBF2. (**A**) Cell number of H295R cells transfected with negative control siRNA (siControl), siRNA targeting to ARMC5 (siARMC5), and/or siRNA targeting to SREBF2 (siSREBF2) (*n* = 3, each). *P < 0.05 by Tukey-Kramer test. (**B**) Gene expression of indicated genes in H295R cells transfected with negative control siRNA (Cont), siRNA targeting to ARMC5 (ARMC5) and/or siRNA targeting to SREBF2 (SREBF2) for 72 hours (*n* = 3, each). *P < 0.05, ***P < 0.001 by Tukey-Kramer test, compared with siControl. (**C**) TUNEL assay in H295R cells transfected with negative control siRNA (siControl) or siRNA targeting to ARMC5 (siARMC5) for 72 hours (*n* = 6, each). The percentage of TUNEL^+^ nuclei was expressed relative to the number of DAPI. Difference was analyzed by 2-tailed *t* test. (**D**) Cell number of H295R-*TetON-hARMC5* (ARMC5[WT]) or H295R-*TetON-hARMC5(R362L)* (ARMC5[R362L]) transfected with negative control siRNA (C) or siRNA targeting to ARMC5 (A) for 48 hours followed by treatment of doxycycline for 48 hours (*n* = 4, each). *P < 0.05 by Tukey-Kramer test. (**E**) Flow cytometry analysis of cell cycle in H295R cells transfected with negative control siRNA (siControl), siRNA targeting to ARMC5 (siARMC5), and/or siRNA targeting to SREBF2 (siSREBF2) (*n* = 6, each). *P < 0.05 by Tukey-Kramer test.

Finally, we investigated the role of ARMC5-SREBF2 interaction on the cell growth of adrenocortical cells. Consistent with the recent report (12), knockdown of *ARMC5* increased cell growth (Figure 7A) with upregulation of *CCND1* and *CCNE1* (Figure 7B and Supplemental Figure 5), while knockdown of *ARMC5* had no effects on the apoptosis, as evidenced by TUNEL assay (Figure 7C). siARMC5-mediated cell growth was abolished by doxycycline-induced overexpression of *ARMC5* in H295R-*TetON-hARMC5* cells but not by that of *ARMC5(R362L)* in H295R-*TetON-hARMC5(R362L)* cells (Figure 7D). The siARMC5-mediated cell growth and upregulation of *CCNE1* were abolished by simultaneous knockdown of *SREBF2* (Figure 7, A and B). Consistent with the recent report (12), *ARMC5* silencing decreased the percentage of cells in G1 phase and increased the percentage of cells in S phase in H295R cells (Figure 7E). Simultaneous knockdown of *SREBF2* abolished these effects, indicating that ARMC5 was involved in cell cycle progression through SREBF2. These findings indicate that endogenous ARMC5 inhibited SREBF2 activity and that SREBF2 was involved in the tumor-suppressor activity of ARMC5 in adrenocortical cells.

## Discussion

In the current study, we identified the interaction between the N-terminus of full-length SREBF and the Armadillo repeat of ARMC5 by biochemical purification (Figure 1). Several interactors of SREBF have been identified to date. In the nucleus, CREBBP (15), EP300 (15), and PPARGC1B (6) interact with nuclear SREBF and function as coactivators. FBXW7 interacts with and degrades nuclear SREBF (16). SCAP interacts with the C-terminus of full-length SREBF and mediates its transport from the ER to the Golgi. In the current study, we reported the interactor with the N-terminus of full-length SREBF.

Biochemical purification also identified CUL3 as an interactor of the BTB domain of ARMC5, and ARMC5 ubiquitinated and degraded itself (Figure 3), which was consistent with the recent report by Cavalcante et al. (12). From the domain structure of ARMC5 (i.e., 2 protein-interacting surfaces without enzymatic domains), it is likely that ARMC5 is an adaptor protein that recruits specific substrates for degradation. For example, the BTB-containing protein KEAP1 is an adaptor between CUL3 and NRF2 that facilitates degradation (21). In fact, ARMC5 effectively degraded full-length SREBF1 through a proteasome-dependent mechanism (Figure 4), and knockdown of endogenous ARMC5 increased full-length SREBFs and gene expression of their target genes (Figure 6). From these data, we concluded that full-length SREBF was a substrate of ARMC5-CUL3 E3 ligase complex.

*ARMC5* is considered a tumor-suppressor gene, as silencing of *ARMC5* led to increased proliferation of adrenocortical cells with upregulation of *CCNE1* (10). In this study, we reveal that siARMC5-mediated proliferation was dependent on *SREBF* with upregulation of cholesterol-related genes (Figure 6 and Figure 7). This is consistent with recent evidence that the SREBF pathway was involved in tumor progression. SREBF was activated and necessary for tumor growth in various tumors, such as glioblastoma (22), prostate cancer (23), breast cancer (24, 25), and colon cancer (26). As for adrenocortical tumor, it was more recently reported that reduction of intracellular cholesterol inhibited adrenocortical cancer growth with suppression of *CCNE1* (27).

Adrenocortical cells are supposed to be highly dependent on SREBF, as *SREBF* represents the highest expression in the adrenal cortex (Supplemental Figure 2) and steroids are synthesized from cellular cholesterol. In the normal adrenocortical cells, steroidogenesis expense cholesterol, and decreased cellular cholesterol activate SREBF to upregulate cholesterol synthesis (28) or uptake (29) and maintain cellular cholesterol. This process would be properly regulated by ARMC5 through degradation of SREBF protein. In the absence of functional ARMC5 in BMAH, steroidogenesis-mediated cholesterol depletion would overactivate SREBF to synthesize/uptake excess cholesterol. Excess cellular cholesterol would, in turn, facilitate cell proliferation, ultimately leading to tumor progression.

The current findings implicated several clinical perspectives. If BMAH were highly dependent on SREBF2 and cholesterol metabolism, administration of statin might inhibit growth of BMAH through depletion of cellular cholesterol. This was intriguing because it is difficult to treat BMAH by surgery, as removal of bilateral tumor in BMAH would result in secondary adrenal insufficiency. As *ARMC5* was ubiquitously expressed, and SREBF plays important roles in metabolism, including diabetes, dyslipidemia, and liver steatosis, ARMC5 might also be an important factor beyond BMAH. Since E3 ligases are potential targets for small molecules (30), ARMC5 might be a candidate for the treatment of metabolic syndrome.

**Figure 8:**
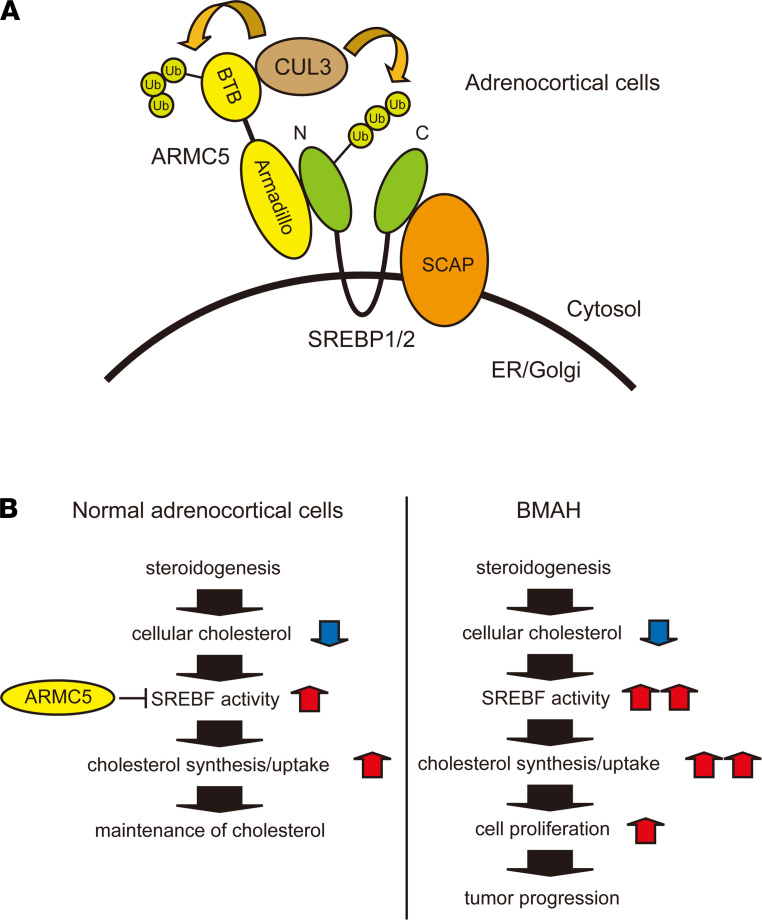
Schematic representation of the possible roles of the SREBF and ARMC5 in adrenocortical cells. (**A**) ARMC5 interacts with the N-terminus of full-length SREBF1/2 through the Armadillo repeat and CUL3 through the BTB domain. The CUL3-ARMC5 E3 complex ubiquitinates and degrades ARMC5 itself and full-length SREBF. (**B**) In the normal adrenocortical cells, steroidogenesis requires cellular cholesterol. Decreased cellular cholesterol activates SREBF and accelerates cholesterol synthesis/uptake to maintain cellular cholesterol. This process is properly regulated by ARMC5 through degradation of SREBF protein (left). In the absence of functional ARMC5 in BMAH, decreased cellular cholesterol by steroidogenesis overactivates SREBF to synthesize or uptake excess cholesterol. Excess cellular cholesterol, in turn, stimulates cell proliferation, ultimately leading to tumor progression (right).

In conclusion, we identified the interaction among ARMC5, SREBF, and CUL3. ARMC5 was a molecular adaptor between full-length SREBF and CUL3-based E3 ligase, and it degraded SREBF protein. SREBF was involved in the tumor-suppressor function of ARMC5 in adrenocortical cells. These findings implicated the mechanisms how the inactivation of ARMC5 leads to BMAH (Figure 8).

## Methods

### Plasmids.

The entire coding sequence of mouse *Srebf1* variant 1 was cloned and inserted into pcDNA3.1-*FLAG* to generate pcDNA3.1-*FLAG-Srebf1*. Base pairs 4–1359 from the start codon of the coding sequence of mouse *Srebf1* variant 1 were cloned and inserted into pcDNA3.1-*FLAG* and pRetroX-Tight-*Pur-FLAG* (Takara Bio) to generate pcDNA3.1-*FLAG-Srebf1(N)* and pRetroX-Tight-*Pur-FLAG-Srebf1(N)*, respectively. Base pairs 4–1377 from the start codon of the coding sequence of mouse *Srebf2* were cloned and inserted into pcDNA3.1-*FLAG* to generate pcDNA3.1-*FLAG-Srebf2(N)*. The full-length coding sequence of mouse *Armc5* was cloned and inserted into pcDNA3.1-*FLAG*, pcDNA3.1-*myc*, and pRetroX-Tight-*Pur-FLAG* to generate pcDNA3.1-*FLAG-mArmc5*, pcDNA3.1-*myc-mArmc5*, and pRetroX-Tight-*Pur-FLAG-Armc5*, respectively. Base pairs 1324–2781 from the start codon of the coding sequence of mouse *Armc5* were cloned and inserted into pcDNA3.1-*myc* to generate pcDNA3.1-*myc-mArmc5ΔARM*. Base pairs 1–2190 from the start codon of the coding sequence of mouse *Armc5* were cloned and inserted into pcDNA3.1-*myc* or pRetroX-Tight-*Pur-FLAG* to generate pcDNA3.1-*myc-mArmc5ΔBTB* or pRetroX-Tight-*Pur-FLAG-mArmc5ΔBTB*, respectively. The full-length coding sequence of human *ARMC5* variant 1 was cloned and inserted into pcDNA3.1-*myc* to generate pcDNA3.1-*myc-hARMC5*. The point mutation C943T, T992C, G1085T, T2261C, or C2692T was introduced into pcDNA3.1-*myc-hARMC5* to generate pcDNA3.1-*myc-hARMC5(R315W)*, pcDNA3.1-*myc-hARMC5(L331P)*, pcDNA3.1-*myc-hARMC5 (R362L)*, pcDNA3.1-*myc-hARMC5(L754P)* or pcDNA3.1-*myc-hARMC5(R898W)*, respectively. Human *ARMC5* or human *ARMC5(R362L)* were inserted into pRetroX-Tight-*Pur-FLAG* to generate pcDNA3.1-*myc-hARMC5* or pRetroX-Tight-*Pur-FLAG-hARMC5(R362L)*, respectively.

### Cell culture.

HEK293T cells and 3T3-L1 mouse fibroblasts were purchased from ATCC and were maintained in DMEM (high glucose) (Nacalai Tesque) supplemented with 10% FBS (Thermo Fisher Scientific) and penicillin/streptomycin (Nacalai Tesque). The 3T3-L1 cells were differentiated into adipocytes by treatment with 2.5 μM dexamethasone (Sigma-Aldrich), 2 μM insulin (Sigma-Aldrich), 0.5 mM 3-isobutyl-1-methylxanthine (Nacalai Tesque), and 1 μM pioglitazone (Sigma-Aldrich) for 2 days. NCI-H295R cells (CRL-2128) were purchased from ATCC and maintained in DMEM/F12 (Thermo Fisher Scientific) supplemented with 1% ITS+ Premix (Corning), 2.5% Nu-Serum (Corning), and penicillin/streptomycin (Nacalai Tesque).

### Retroviral infection.

Platinum-E cells were transfected with pRetroX-Tet-On Advanced (Takara Bio), pRetroX-Tight-*Pur-FLAG*, pRetroX-Tight-*Pur-FLAG-Srebf1(N)*, pRetroX-Tight-*Pur-FLAG-Armc5*, or pRetroX-Tight-*Pur-FLAG-Armc5ΔBTB*. Forty-eight hours after transfection, the media containing the ecotropic retroviruses were harvested, filtered, and transferred to 3T3-L1 cells using 10 μg/mL polybrene (Sigma-Aldrich). Infected cells were selected using 800 μg/mL G418 (Nacalai Tesque) and 2 μg/mL puromycin (Nacalai Tesque). The resultant cells were referred to as 3T3-L1–*TetON-FLAG*, 3T3-L1–*TetON-FLAG-Srebf1(N)*, 3T3-L1–*TetON-FLAG-Armc5*, or 3T3-L1–*TetON-FLAG-Armc5ΔBTB*. Pantropic retrovirus were obtained using the Retro-X Universal Packaging system (Takara) with pRetroX-Tight-*Pur-FLAG-hARMC5* or pRetroX-Tight-*Pur-FLAG-hArmc5(R362L)*. These viruses were transferred to H295R cells without polybrene. Infected cells were selected using 800 μg/mL of G418 and 5 μg/mL of puromycin. The resultant cells were referred to as H295R-*TetON-FLAG-hARMC5* or H295R-*TetON-FLAG-hARMC5(R362L)*, respectively.

### Western blotting and immunoprecipitation.

Cells were lysed with TNE buffer (10 mM Tris-HCl [Nacalai Tesque], 150 mM NaCl, 1 mM EDTA [Nacalai Tesque], 1% NP40 [Nacalai Tesque], and 1/100 Proteinase Inhibitor Cocktail [Nacalai Tesque, 25955-11]) and subjected to Western blotting with antibodies. The antibodies used were anti–FLAG M2-HRP (Sigma-Aldrich, A8592), anti-myc antibody (9B11) (HRP conjugate) (Cell Signaling Technology, 2040), anti-CUL3 antibody (Abcam, ab75851), anti-ACTB (Sigma-Aldrich, A5441), anti-SREBF1 (2A4) (Santa Cruz, sc-13551), anti-SREBF2 (R&D Systems, MAB7119), and anti-ARMC5 (Novus Biologicals, NBP1-94024). For immunoprecipitation of FLAG-tagged protein, the cells were immunoprecipitated using anti-FLAG M2 Affinity Gel (Sigma-Aldrich), washed with TNE buffer, and eluted with 200 μg/mL FLAG peptide (Sigma-Aldrich, F3290). For immunoprecipitation of Myc-tagged protein, the cells were incubated with anti-myc antibody (Cell Signaling Technology, 2276), immunoprecipitated with Protein G Sepharose 4 Fast Flow (GE Healthcare, 17-0618-01), washed with TNE buffer, and boiled in sample buffer. In the coimmunoprecipitation of ARMC5 and CUL3, HEK293T cells were lysed with high-salt TNE buffer (10 mM Tris-HCl, 350 mM NaCl, 1 mM EDTA, 1% NP40, and 1/100 Proteinase Inhibitor Cocktail [Nacalai Tesque]), immunoprecipitated using anti-FLAG M2 Affinity Gel (Sigma-Aldrich), and eluted with 200 μg/mL FLAG peptide (Sigma-Aldrich).

### Biochemical purification.

Nuclear pellets from thirty 15 cm culture dishes of differentiated 3T3-L1 cells were prepared by a Dounce homogenizer (Wheaton) using hypotonic buffer (10 mM HEPES-KOH, 1.5 mM MgCl_2_, and 10 mM KCl [Nacalai Tesque]). Nuclear protein was extracted with low-salt buffer (20 mM HEPES-KOH, 0.2 mM EDTA, 1.5 mM MgCl_2_ [Nacalai Tesque], and 25% glycerol), followed by the addition of 4M NaCl. Extracted protein was dialyzed with dialysis buffer (20 mM HEPES-KOH, 150 mM KCl, 0.2 mM EDTA, 10% glycerol [Nacalai Tesque], and 0.05% NP40). Extracts were immunoprecipitated using anti-FLAG M2 Affinity Gel (Sigma-Aldrich) and eluted with 200 μg/mL FLAG peptide (Sigma-Aldrich). Samples were subjected to LC-MS/MS using an UltiMate 3000 Nano LC system, Q-Exactive (Thermo Fisher Scientific).

### Immunofluorescence staining.

Differentiated 3T3-L1 adipocytes or H295R cells on Cell Desk LF1 (Sumitomo Bakelite) were fixed with 4% paraformaldehyde for 10 minutes and permeabilized with 0.1% Triton X-100 in phosphate-buffered saline (PBS). The cells were incubated with 10% goat serum for 30 minutes and incubated overnight at 4°C with anti-ARMC5 antibody (Novus Biologicals, NBP1-94024) conjugated to Texas Red and anti-SREBF1 antibody (Abcam, ab28481), conjugated to DyLight 488. Conjugation was performed using a Texas Red Conjugation Kit (Fast) (Abcam, ab195225) or DyLight 488 Conjugation Kit (Abcam, ab201799) according to the manufacturer’s instructions. Microscopy was performed using an FV1000D confocal laser scanning microscope system (Olympus).

### In situ proximity ligation assay.

In situ proximity ligation assay was performed using Duolink in situ starter set RED (Sigma Aldrich), according to the manufacturer’s protocol. H295R cells on Cell Desk LF1 (Sumitomo Bakelite) were fixed with 4% paraformaldehyde (Nacalai Tesque) for 10 minutes and permeabilized with 0.1% Triton X-100 in PBS. The cells were incubated with Duolink Blocking Solution and incubated with anti-ARMC5 antibody (Novus Biologicals, NBP1-94024) and anti-SREBF1 antibody (Proteintech, 66875-1-Ig). Microscopy was performed using an FV1000D confocal laser scanning microscope system (Olympus).

### Cell-based ubiquitination assay.

For detection of ubiquitinated ARMC5 in vivo, HEK293T cells or H295R cells were transfected with pRK5-HA-Ubiquitin-WT (Addgene, 17608) (31) and pcDNA3.1-*FLAG-mArmc5*. For detection of full-length SREBF1 in vivo, HEK293T cells were transfected with pRK5-HA-Ubiquitin-WT and pcDNA3.1-*FLAG-Srebf1* with pcDNA3.1-*myc* or pcDNA3.1-*myc-Armc5*. Twenty-four hours after transfection, the cells were treated with 10 μM MG132 (Sigma-Aldrich) for 5 hours. The cells were lysed by boiling in a buffer containing 2% SDS, 150 mM NaCl, 10 mM Tris-HCl, and 1 mM DTT (Nacalai Tesque). These lysates were diluted ninefold in dilution buffer containing 150 mM NaCl, 10 mM Tris-HCl, and 1% Triton X-100 and immunoprecipitated with anti-FLAG M2 Affinity Gel (Sigma-Aldrich); washed 4 times with dilution buffer; eluted with 200 μg/mL FLAG peptide (Sigma-Aldrich); and subjected to Western blotting using rabbit anti-HA antibody (Cell Signaling Technology, 3724) and HRP-conjugated anti-rabbit IgG antibody (GE Healthcare, NA934V).

### Transfection of siRNA.

Silencer Select siRNAs (12 pmol) targeting to *ARMC5* (Thermo Fisher Scientific, s36352), *SREBF2* (Thermo Fisher Scientific, s29) or Silencer Select Negative Control No.1 siRNA (Thermo Fisher Scientific) were introduced to NCI-H295R cells by reverse transfection using 3 μL of Lipofectamine RNAiMAX Reagent (Thermo Fisher Scientific) per 12-well plate according to the protocol provided by the manufacturer. Silencer Select siRNA (180 pmol) targeting to *CUL3* (Thermo Fisher Scientific, s16050) or Silencer Select Negative Control No.1 siRNA (Thermo Fisher Scientific) were introduced to HEK293T cells using 30 μL of Lipofectamine RNAiMAX Reagent (Thermo Fisher Scientific) per 10 cm dish according to the protocol provided by the manufacturer.

### mRNA analysis.

Total RNA was isolated by TRI Reagent (Sigma-Aldrich) according to the protocol provided by the manufacturer. First-strand cDNA was synthesized from total RNA using the Transcriptor First Strand cDNA Synthesis Kit (Roche). cDNA was subjected to quantitative PCR (qPCR) using a LightCycler system (Roche) according to the instructions provided by the manufacturer. The mRNA expression levels were measured relative to those of *RPLP0*. Relative mRNA expression is the value calculated relative to standard samples in real-time PCR. The primers used in this procedure are shown in Table 1.

### Proliferation assays.

A total of 70,000 NCI-H295R cells, H295R-*TetON-hARMC5* cells, or H295R-*TetON-hARMC5(R362L)* cells were transfected with siRNAs by reverse transfection using Lipofectamine RNAiMAX onto 12-well plates. Twenty-four, 48, 72, or 96 hours after transfection, the cells were trypsinized and resuspended in 1 mL of growth medium. Cell numbers were acquired using TC20 automated cell counter (Bio-Rad).

### TUNEL assay.

TUNEL assay was performed using In situ Apoptosis Detection Kit (Takara Bio) according to the manufacturer’s protocol. H295R cells on Cell Desk LF1 (Sumitomo Bakelite) were fixed with 4% paraformaldehyde for 10 minutes and permeabilized with Permeabilisation Buffer (Takara Bio, MK505) for 15 minutes. Microscopy was performed using an BZ9000 fluorescence microscopy (Keyence).

### Cell cycle analysis.

Cells were harvested using trypsin/EDTA and fixed in 70% ethanol. Fixed cells were washed with PBS and resuspended in solution containing 20 μg/mL of propidium iodide (Fujifilm Wako), 200 μg/mL of RNase A (Nacalai Tesque), and 0.1% Triton X-100. Cells were analyzed by flow cytometry using SH800ZDP (Sony). Data were analyzed using FlowJo.

### Statistics.

Data are presented as the mean ± SD. Differences between 2 groups were analyzed by 2-tailed *t* tests. Differences among multiple groups were analyzed by Tukey-Kramer tests or Dunnett’s tests using JMP Pro 12. Significance was set at *P* < 0.05.

## Author contributions

YO designed the research protocol, performed experiments, analyzed the data, and cowrote the manuscript. AF, MO, and IS directed the research and cowrote the manuscript. All authors discussed and interpreted the data.

## Supplementary Material

Supplemental data

## Figures and Tables

**Table 1:**
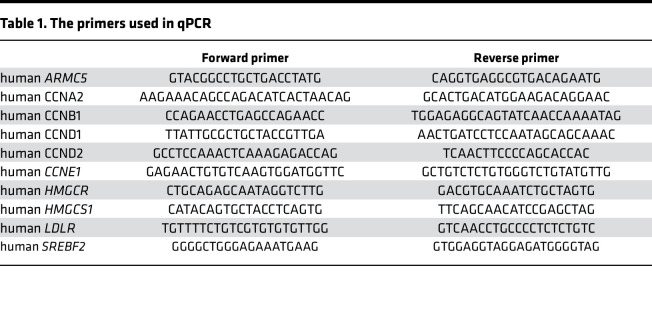
The primers used in qPCR

## Version 1 07/21/2022

In-Press Preview

## Version 2 08/22/2022

Electronic publication

## References

[1] Vaduva P (2020). Molecular basis of primary aldosteronism and adrenal Cushing syndrome. J Endocr Soc.

[2] Beuschlein F (2014). Constitutive activation of PKA catalytic subunit in adrenal Cushing’s syndrome. N Engl J Med.

[3] Cao Y (2014). Activating hotspot L205R mutation in PRKACA and adrenal Cushing’s syndrome. Science.

[4] Goh G (2014). Recurrent activating mutation in PRKACA in cortisol-producing adrenal tumors. Nat Genet.

[5] Sato Y (2014). Recurrent somatic mutations underlie corticotropin-independent Cushing’s syndrome. Science.

[6] Kirschner LS (2000). Mutations of the gene encoding the protein kinase A type I-alpha regulatory subunit in patients with the Carney complex. Nat Genet.

[7] Kobayashi H (2000). Mutation analysis of Gsalpha, adrenocorticotropin receptor and p53 genes in Japanese patients with adrenocortical neoplasms: including a case of Gsalpha mutation. Endocr J.

[8] Horvath A (2006). A genome-wide scan identifies mutations in the gene encoding phosphodiesterase 11A4 (PDE11A) in individuals with adrenocortical hyperplasia. Nat Genet.

[9] Assie G (2013). ARMC5 mutations in macronodular adrenal hyperplasia with Cushing’s syndrome. N Engl J Med.

[10] Cavalcante IP (2018). The role of ARMC5 in human cell cultures from nodules of primary macronodular adrenocortical hyperplasia (PMAH). Mol Cell Endocrinol.

[11] Drougat L (2015). Novel insights into the genetics and pathophysiology of adrenocortical tumors. Front Endocrinol (Lausanne).

[12] Cavalcante IP (2020). Cullin 3 targets the tumor suppressor gene ARMC5 for ubiquitination and degradation. Endocr Relat Cancer.

[13] DeBose-Boyd RA, Ye J (2018). SREBPs in lipid metabolism, insulin signaling, and beyond. Trends Biochem Sci.

[14] Okuno Y (2018). Oxidative stress inhibits healthy adipose expansion through suppression of SREBF1-mediated lipogenic pathway. Diabetes.

[15] Oliner JD (1996). SREBP transcriptional activity is mediated through an interaction with the CREB-binding protein. Genes Dev.

[16] Sundqvist A (2005). Control of lipid metabolism by phosphorylation-dependent degradation of the SREBP family of transcription factors by SCF(Fbw7). Cell Metab.

[17] Hu Y (2017). Armc5 deletion causes developmental defects and compromises T-cell immune responses. Nat Commun.

[18] Berthon A (2017). Age-dependent effects of Armc5 haploinsufficiency on adrenocortical function. Hum Mol Genet.

[19] Pintard L (2004). Cullin-based ubiquitin ligases: Cul3-BTB complexes join the family. EMBO J.

[20] Dorotea D (2020). Recent insights into SREBP as a direct mediator of kidney fibrosis via lipid-independent pathways. Front Pharmacol.

[21] Canning P (2015). Structural basis of Keap1 interactions with Nrf2. Free Radic Biol Med.

[22] Guo D (2009). EGFR signaling through an Akt-SREBP-1-dependent, rapamycin-resistant pathway sensitizes glioblastomas to antilipogenic therapy. Sci Signal.

[23] Huang WC (2012). Activation of androgen receptor, lipogenesis, and oxidative stress converged by SREBP-1 is responsible for regulating growth and progression of prostate cancer cells. Mol Cancer Res.

[24] Bao J (2016). SREBP-1 is an independent prognostic marker and promotes invasion and migration in breast cancer. Oncol Lett.

[25] Ricoult SJ (2016). Oncogenic PI3K and K-Ras stimulate de novo lipid synthesis through mTORC1 and SREBP. Oncogene.

[26] Wen YA (2018). Downregulation of SREBP inhibits tumor growth and initiation by altering cellular metabolism in colon cancer. Cell Death Dis.

[27] Trotta F (2020). Statins reduce intratumor cholesterol affecting adrenocortical cancer growth. Mol Cancer Ther.

[28] Rainey WE (1986). ACTH induction of 3-hydroxy-3-methylglutaryl coenzyme A reductase, cholesterol biosynthesis, and steroidogenesis in primary cultures of bovine adrenocortical cells. J Biol Chem.

[29] Martin G (1999). Comparison of expression and regulation of the high-density lipoprotein receptor SR-BI and the low-density lipoprotein receptor in human adrenocortical carcinoma NCI-H295 cells. Eur J Biochem.

[30] Bulatov E (2018). Small molecule modulators of RING-type E3 ligases: MDM and cullin families as targets. Front Pharmacol.

[31] Lim KL (2005). Parkin mediates nonclassical, proteasomal-independent ubiquitination of synphilin-1: implications for Lewy body formation. J Neurosci.

