# Supplementary material for: ARMC5-CUL3 E3 ligase targets full-length SREBF in adrenocortical tumors
Source: JCI Insight. 2022 Aug 22;7(16):e151390. doi: 10.1172/jci.insight.151390 (PMC9462479; doi:10.1172/jci.insight.151390)
Supplement: Supplemental data [file jciinsight-7-151390-s142.pdf]

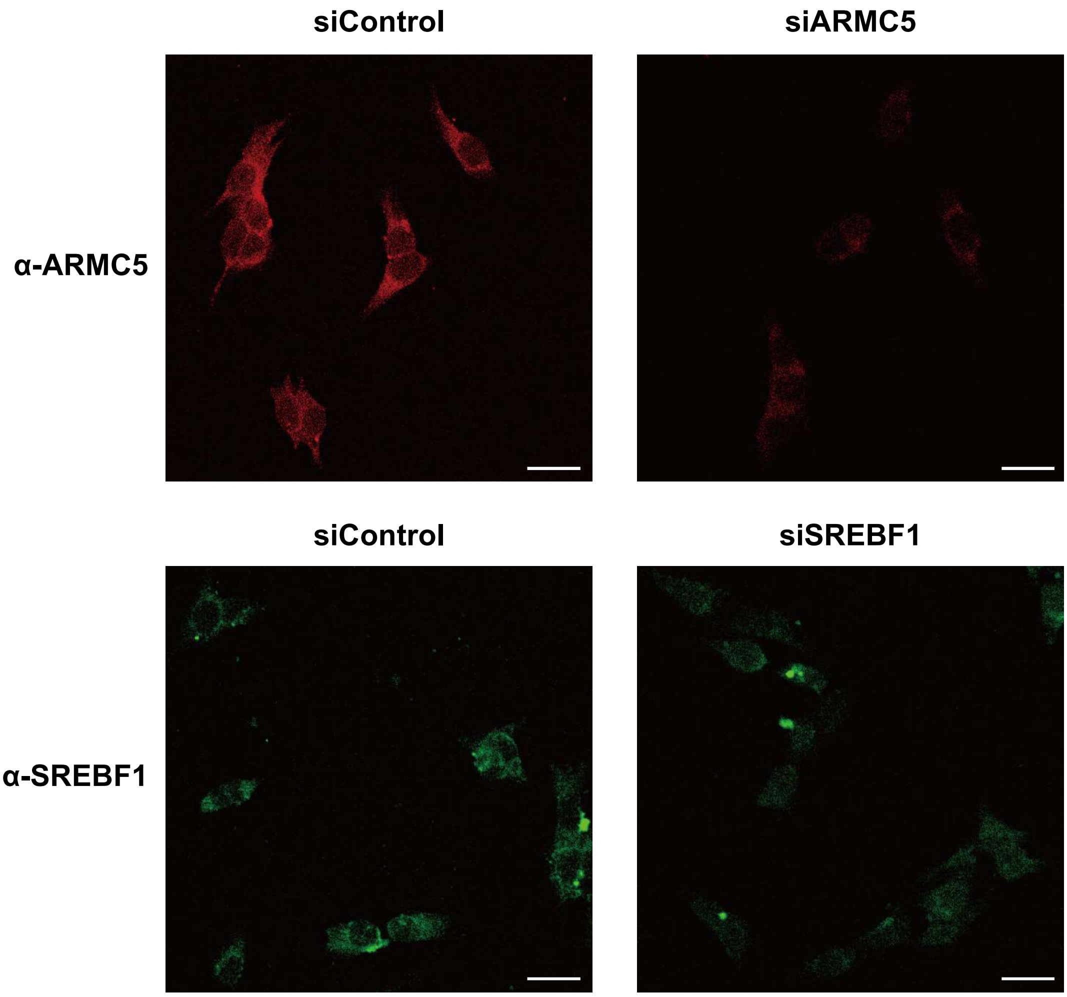


Supplementary Figure 1. Confocal microscopy of NCI-H295R adrenocortical cells transfected with negative control siRNA (siControl), siRNA targeting to ARMC5 (siARMC5) or SREBF1 (siSREBF1) for 48 hours, stained with Texas Red-anti-ARMC5 (red) or DyLight488-anti-SREBF1 (green). Scale bar; 20 μm.

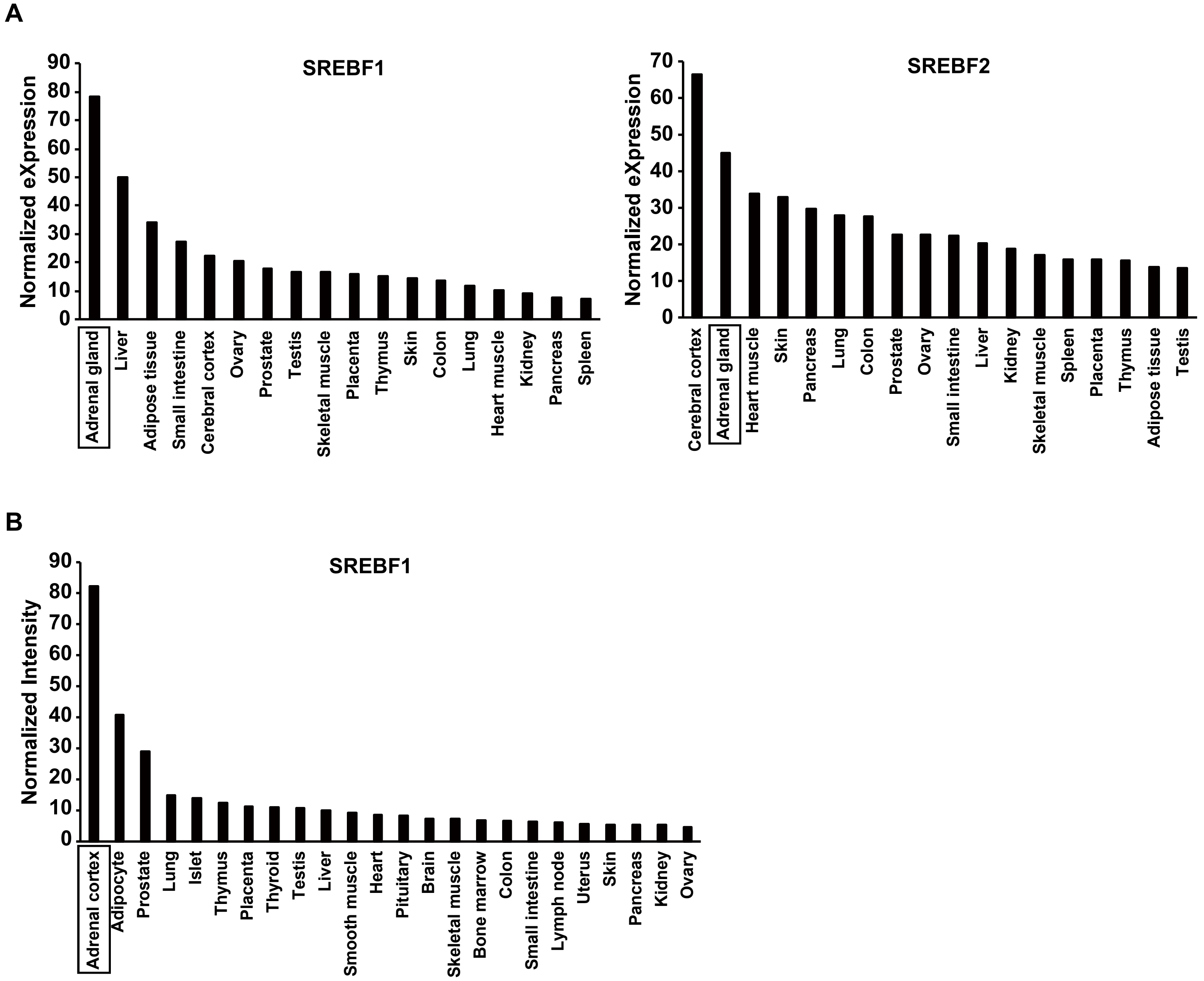


Supplementary Figure 2. A: RNA expression overview of the Consensus dataset from The Human Protein Atlas (www.proteinatlas.org) of the indicated genes in the indicated human tissues. B: Gene expression/activity chart from BioGPS (www.biogps.org) of SREBF1 in the indicated human tissues.

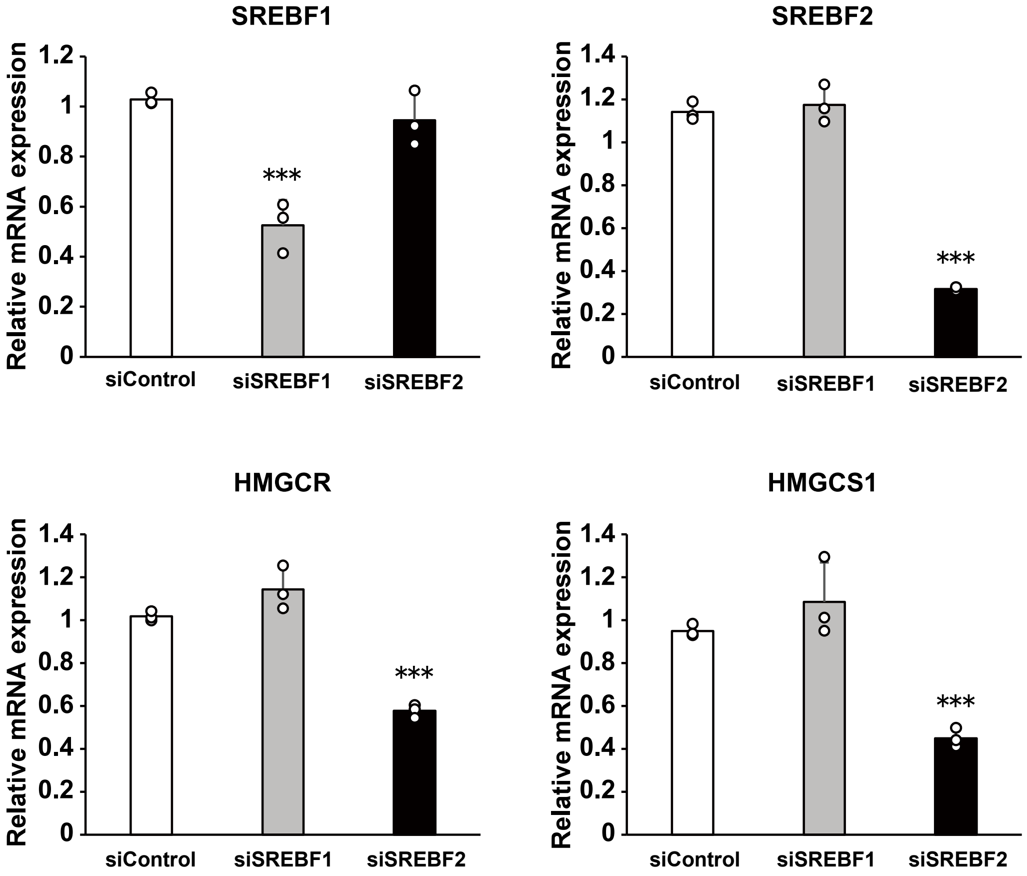


Supplementary Figure 3. Gene expression of indicated genes in H295R cells transfected with negative control siRNA (siControl) , siRNA targeting to SREBF1 (siSREBF1) or SREBF2 (siSREBF2) for 48 hours (n=3, each). ***P<0.001, compared with siControl.

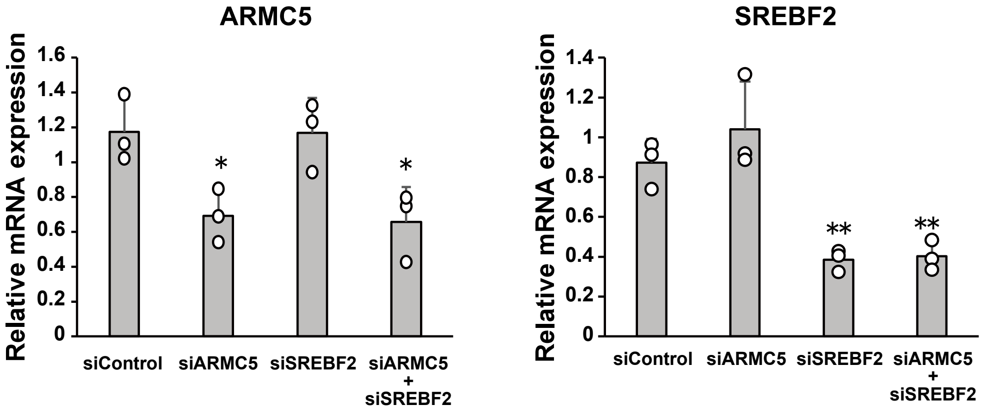


Supplementary Figure 4. Gene expression of the indicated genes in H295R cells transfected with negative control siRNA (siControl), siRNA targeting to ARMC5 (siARMC5) and/or siRNA targeting to SREBF2 (siSREBF2) for 72 hours (n=3, each). *P<0.05; **P<0.01, compared with siControl.

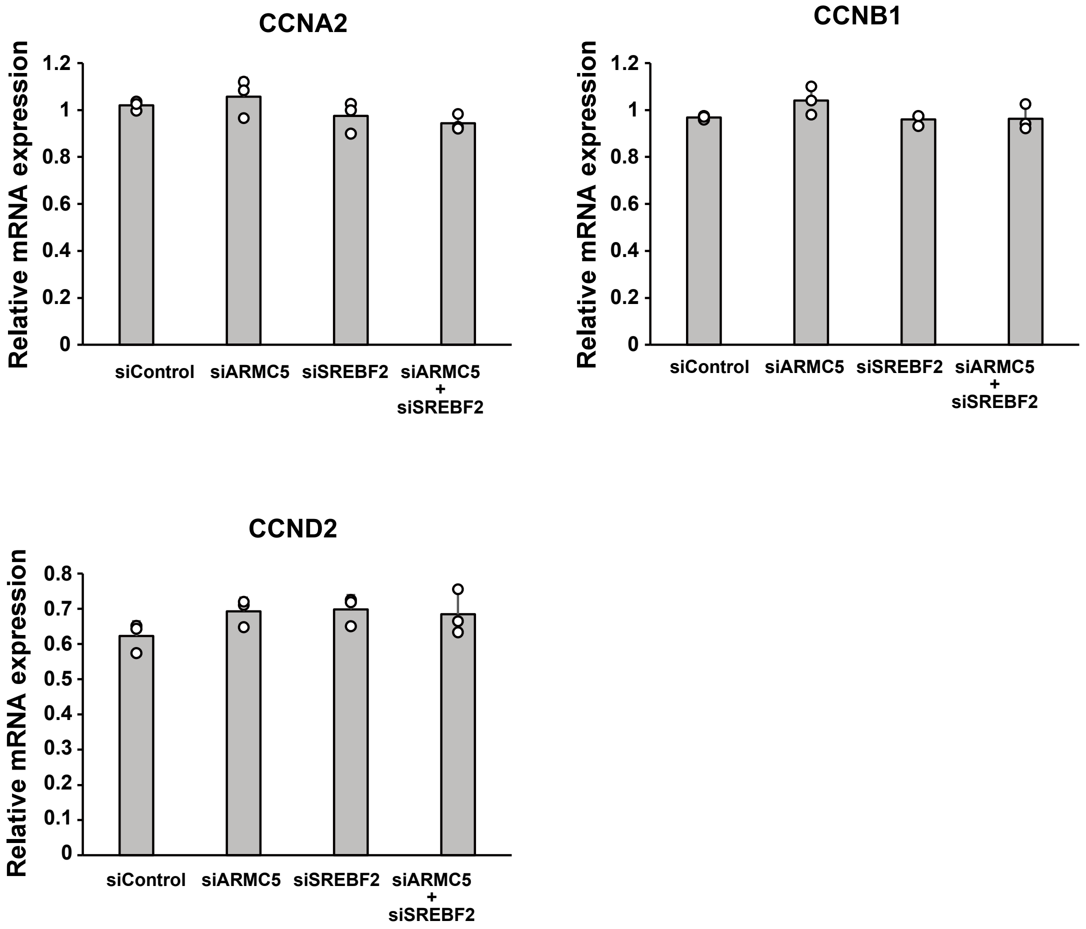


Supplementary Figure 5. Gene expression of the indicated genes in H295R cells transfected with negative control siRNA (siControl), siRNA targeting to ARMC5 (siARMC5) and/or siRNA targeting to SREBF2 (siSREBF2) for 72 hours (n=3, each).
